# Study Design and Baseline Profiles of Participants in the Uonuma CKD Cohort Study in Niigata, Japan

**DOI:** 10.2188/jea.JE20180220

**Published:** 2020-04-05

**Authors:** Keiko Kabasawa, Junta Tanaka, Kazutoshi Nakamura, Yumi Ito, Kinya Yoshida, Ribeka Takachi, Norie Sawada, Shoichiro Tsugane, Ichiei Narita

**Affiliations:** 1 Department of Health Promotion Medicine, Niigata University Graduate School of Medical and Dental Sciences, Niigata, Japan; 2 Division of Preventive Medicine, Niigata University Graduate School of Medical and Dental Sciences, Niigata, Japan; 3 Department of Food Science and Nutrition, Nara Women’s University Graduate School of Humanities and Sciences, Nara, Japan; 4 Epidemiology and Prevention Group, Center for Public Health Sciences, National Cancer Center, Tokyo, Japan; 5 Division of Clinical Nephrology and Rheumatology, Niigata University Graduate School of Medical and Dental Sciences, Niigata, Japan

**Keywords:** chronic kidney disease, cohort studies, lifestyle, risk factors

## Abstract

**Background:**

Evidence for primary prevention of chronic kidney disease (CKD) is insufficient. The population-based prospective Uonuma CKD cohort study aims to explore associations of lifestyle and other risk factors with CKD. We report here the study design and baseline profiles.

**Methods:**

All 67,322 residents aged ≥40 years in Minamiuonuma City, Uonuma City, and Yuzawa Town, Niigata Prefecture, Japan and 11,406 participants who attended local health-check examinations were targeted for baseline questionnaire and biochemical sampling, respectively. Information was gathered from 43,217 (64.2%) questionnaires and 8,052 (70.6%) biochemical samples; 6,945 participants consented to both questionnaire and biochemical sampling at baseline, conducted between fiscal years 2012 and 2015. Participants provided information regarding sociodemographic, lifestyle, and self-reported outcomes. Urine albumin-to-creatinine ratio (ACR) and estimated glomerular filtration rate (eGFR) were measured. The primary outcome is CKD based on self-report and biochemical/clinical diagnosis.

**Results:**

Mean age of questionnaire respondents was 63.3 (standard deviation [SD], 12.5) years for men and 64.3 (SD, 13.3) years for women. Among participants who submitted urine samples, median ACR was 10.0 (interquartile range [IQR], 5.0–24.0) mg/g for men and 13.0 (IQR, 7.7–27.0) mg/g for women, and median eGFR was 73.6 mL/min/1.73 m^2^ (IQR, 63.5–84.5) for men and 73.5 mL/min/1.73 m^2^ (IQR, 64.4–83.5) for women. ACR 30 mg/g or more was found in 1,741 participants (21.7%) and eGFR <60 mL/min/1.73 m^2^ in 1,361 participants (16.9%).

**Conclusion:**

The Uonuma CKD cohort study was established to investigate the impact of lifestyle on CKD development and to provide data for preventing the onset and progression of CKD.

## INTRODUCTION

Chronic kidney disease (CKD) is defined by the presence of urine or kidney abnormalities or low glomerular filtration rate (GFR).^1^ CKD promotes end-stage renal disease and is a risk factor for cardiovascular disease^2^ and all-cause mortality.^3^^,^^4^ CKD is attracting increasing attention because of these adverse outcomes.^5^ The prevalence of CKD is reported as 10–16% of the adult population worldwide.^6^ There are 13.3 million Japanese adults with CKD (12.9%),^7^ and there is concern that the prevalence of end-stage CKD will rise with population aging.

Previous epidemiological studies have tended to focus on secondary prevention of CKD, and current guidelines for CKD are focused mainly on preventing progression to end-stage renal disease. There are several cohort studies of CKD in adult populations, including the Chronic Renal Insufficiency Cohort (CRIC) study in the United States^8^ and the Korean Cohort Study for Outcome in Patients With Chronic Kidney Disease (KNOW-CKD).^9^ Although these studies are large in scale—including 2,500–4,000 patients with CKD—they are hospital-based studies, not population-based studies, that are focusing on comprehensive prevention, including primary prevention, of CKD.

Although CKD is associated with clinical risk factors, such as diabetes and impaired glucose tolerance, hypertension, obesity, and hyperuricemia,^10^^–^^13^ interactions among these clinical factors have not been fully determined. Furthermore, the relationships of various lifestyle factors with the development of CKD remain unclear.

This prospective cohort study aimed to clarify lifestyle-related and clinical risk factors for CKD as well as their interactions, all of which could provide data to support the development of guidelines for primary and secondary prevention of CKD. Here, we describe the study design and present the baseline profiles of participants in this study.

## MATERIAL AND METHODS

### Study design and participants

**Figure 1.**
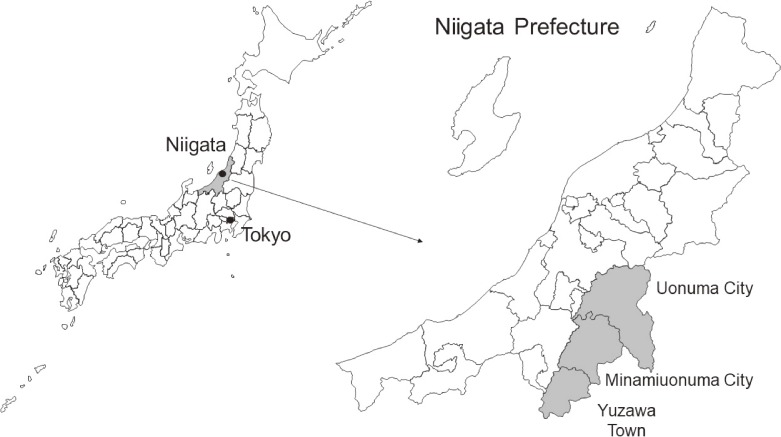


This population-based prospective cohort study has been ongoing since 2012 in the Uonuma area (Minamiunouma City, Uonuma City, and Yuzawa Town) of Niigata Prefecture, Japan. The cohort areas cover the southern part of the prefecture (Figure 1), are characterized by having deep snow almost every winter, and are among the leading producers of Japanese rice.

**Figure 2.**
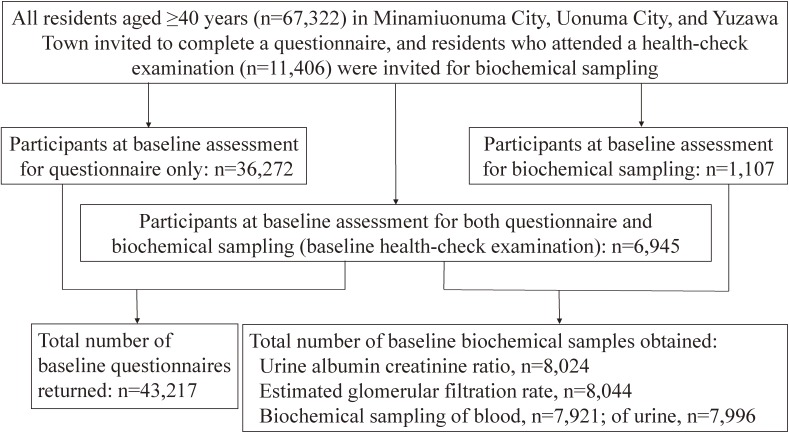


The Uonuma CKD cohort study enrollment flow chart is shown in Figure 2. The target population included residents aged 40 years or older. At baseline, all 67,322 residents of the cohort areas were invited to complete a questionnaire, with consent received from 43,217 residents (64.2%), and all 11,406 residents who attended local health checkups were invited to participate in biochemical sampling, with consent received from 8,052 (70.6%). Consent for both the questionnaire and biochemical sampling (health-check examination) was received from 6,945 participants.

### Procedure

Baseline assessment, comprising the questionnaire and biochemical sampling, was conducted from fiscal year 2012 through 2015: Minamiuonuma City in fiscal years 2012–2013, Uonuma City in fiscal year 2014, and Yuzawa Town in fiscal year 2015.

Self-administered questionnaires in sealed envelopes were hand-distributed to targeted individuals in cooperation with the administrative district managers of Minamiuonuma and Uonuma and by a neighborhood association representative for Yuzawa. Some questionnaires were mailed subsequently. The questionnaire (outlined in Table 1) was based on that used in the Japan Public Health Center-based Prospective Study for the Next Generation (JPHC-NEXT),^14^ comprising approximately 130 questions on demographic characteristics, anthropometrics, disease history, other health information, lifestyle, psychological status, validated physical activity^15^ (including the International Physical Activity Questionnaire^16^^,^^17^), environmental exposures, and a validated 172-item Food Frequency Questionnaire.^18^ Smoking habits and alcohol consumption were determined from questions used in JPHC-NEXT.

**Table 1.** Outline of the baseline self-administered questionnaire

Scope	
Demographics	Age, sex, marital status, household composition, education, occupation, household income
Anthropometrics	Height, weight (current and at 20 years old), waist circumference, birth weight
Health information	Disease history, family medical history, current medications, status of medical examination, dental health information, chronic pain, disability status
Lifestyle	Smoking status, drinking habits, alcohol consumption, sleeping patterns, eating patterns, food preference, internet usage habits, bathing habits (for Yuzawa cohort only)
Psychological status	Self-efficacy, quality of life, work-life balance, current feeling
Physical activity	Sitting, standing, walking, vigorous daily activity time, International Physical Activity Questionnaire, duration and intensity of snow clearing and farming time
Nutrition	Food Frequency Questionnaire of 172 food items for calculating intakes of energy, 53 nutrients, and 29 food groups
Environmental exposure	International Physical Activity Questionnaire Environmental Module (for Uonuma cohort only)
Reproductive (for female participants only)	Menstrual history, pregnancy and birth histories, breastfeeding history, history of hormone usage

### Baseline blood collection and measurements

Participants who attended health-check examinations provided by local governments were asked to consent to provide their health checkup data and blood and urine samples in the year that the baseline assessment was conducted. The unique measurement items of this study were urine albumin-to-creatinine ratio (ACR), urine creatinine, urine sodium, and urine urea nitrogen. For the Yuzawa cohort (the participants from Yuzawa Town) only, serum albumin, serum 25-hydroxyvitamin D, serum cystatin C, urine protein, and grip strength were measured.

Body mass index (BMI) was calculated as body weight (kg) divided by height squared (m^2^); for this calculation, self-reported body weight and height were available for questionnaire participants, and measured values were available for biochemical sampling participants. For the BMI data in Table 2, participants with extremely high or low self-reported BMI (3 standard deviations [SDs] from the mean self-reported BMI for each sex) were excluded from the calculations. Fasting or nonfasting status at the time of blood sample collection was recorded. For the Uonuma cohort (Minamiuonuma City and Uonuma City), blood samples were dispensed into two tubes: EDTA 2Na-containing tubes for plasma separation (7 mL) and serum separating agent-containing tubes (5 mL). Ultimately, 3 mL plasma and 2 mL serum were obtained from each participant. Specimen treatment was performed according to procedures detailed in the JPHC-NEXT study protocol.^14^ Blood samples were stored at −80°C until analysis. For the Yuzawa cohort, blood samples were dispensed into serum separating agent-containing tubes and 5 mL serum was obtained from each participant. One mL serum was used for the measurements; the remaining 4 mL was stored as 0.5-mL aliquots at −80°C on the day of collection. Spot urine samples were collected in the morning or afternoon and were immediately transported to a certified clinical laboratory for analysis. The remaining 5 mL from the Uonuma cohort and 10 mL from the Yuzawa cohort was centrifuged at 3,200 rpm and stored as 1-mL aliquots at −80°C on the same day.

**Table 2.** Selected baseline characteristics based on self-reported questionnaires, by age and sex

	Men Total	Age at baseline, years					Women Total	Age at baseline, years				
	
		40–49	50–59	60–69	70–79	≥80		40–49	50–59	60–69	70–79	≥80
*N* (consent/invited population)	20,559/32,169	3,303/6,097	4,603/7,341	6,231/8,758	4,061/5,630	2,361/4,343	22,658/35,153	3,596/5,932	4,934/6,975	6,063/8,003	4,602/6,395	3,463/7,848
Response rate (%)	63.9	54.2	62.7	71.1	72.1	54.4	64.5	60.6	70.7	75.8	72.0	44.1
Residence area at baseline, *n* (%)												
Minamiuonuma city	10,853 (52.8)	1,783 (54.0)	2,532 (55.0)	3,235 (51.9)	2,107 (51.9)	1,196 (50.7)	11,939 (52.7)	1,959 (54.5)	2,703 (54.8)	3,217 (53.1)	2,354 (51.2)	1,706 (49.3)
Uonuma city	8,040 (39.1)	1,251 (37.9)	1,759 (38.2)	2,472 (39.7)	1,621 (39.9)	937 (39.7)	8,931 (39.4)	1,375 (38.2)	1,901 (38.5)	2,359 (38.9)	1,854 (40.3)	1,442 (41.6)
Yuzawa town	1,666 (8.1)	269 (8.1)	312 (6.8)	524 (8.4)	333 (8.2)	228 (9.7)	1,788 (7.9)	262 (7.3)	330 (6.7)	487 (8.0)	394 (8.6)	315 (9.1)
Conserved blood samples^a^, *n*	3,347	182	303	1,057	1,296	509	3,482	170	341	1,271	1,292	408
Conserved urine samples, *n*	3,363	182	304	1,064	1,302	511	3,538	173	346	1,290	1,312	417
Body mass index^b^, *n* (%)												
<18.5 kg/m^2^	987 (4.9)	100 (3.1)	161 (3.5)	229 (3.7)	233 (5.8)	264 (11.5)	2,265 (10.2)	408 (11.5)	485 (9.9)	515 (8.6)	372 (8.3)	485 (14.7)
18.5–24.9 kg/m^2^	14,694 (72.2)	2,208 (67.4)	3,245 (71.0)	4,503 (72.7)	2,973 (74.2)	1,765 (76.7)	15,916 (71.5)	2,555 (72.1)	3,571 (72.9)	4,345 (72.2)	3,165 (70.2)	2,280 (69.3)
≥25.0 kg/m^2^	4,666 (22.9)	967 (29.5)	1,166 (25.5)	1,459 (23.6)	801 (20.0)	273 (11.9)	4,073 (18.3)	579 (16.4)	840 (17.2)	1,156 (19.2)	973 (21.6)	525 (16)
Educational level, *n* (%)												
Junior high school	6,765 (35.4)	289 (8.9)	721 (16.1)	2,371 (39.0)	2,394 (61.2)	990 (70.6)	8,200 (40.1)	168 (4.7)	590 (12.2)	2,641 (44.7)	3,396 (77.1)	1,405 (81.4)
High school	8,066 (42.2)	1,755 (54.2)	2,380 (53.1)	2,573 (42.3)	1,121 (28.6)	237 (16.9)	7,801 (38.2)	1,955 (55.1)	2,606 (53.9)	2,273 (38.5)	756 (17.2)	211 (12.2)
Junior college	2,253 (11.8)	697 (21.5)	743 (16.6)	554 (9.1)	161 (4.1)	98 (7.0)	3,788 (18.5)	1,208 (34.1)	1,413 (29.2)	862 (14.6)	214 (4.9)	91 (5.3)
University or higher	2,039 (10.7)	495 (15.3)	642 (14.3)	587 (9.7)	238 (6.1)	77 (5.5)	639 (3.1)	217 (6.1)	228 (4.7)	134 (2.3)	41 (0.9)	19 (1.1)
Household income, *n* (%)												
0–2,990,000 yen	6,583 (35.1)	621 (19.5)	945 (21.3)	2,433 (41.0)	1,720 (48.7)	864 (51.9)	7,287 (40.8)	1,026 (30.4)	1,404 (30.8)	2,277 (43.7)	1,605 (53.9)	975 (56.1)
3,000,000–5,990,000 yen	7,928 (42.3)	1,728 (54.3)	1,978 (44.6)	2,384 (40.1)	1,300 (36.8)	538 (32.3)	6,558 (36.7)	1,424 (42.2)	1,722 (37.8)	2,009 (38.5)	948 (31.9)	455 (26.2)
6,000,000–8,990,000 yen	2,748 (14.7)	597 (18.8)	998 (22.5)	700 (11.8)	310 (8.8)	143 (8.6)	2,571 (14.4)	660 (19.6)	876 (19.2)	581 (11.1)	268 (9.0)	186 (10.7)
≥9,000,000 yen	1,491 (8.0)	236 (7.4)	515 (11.6)	422 (7.1)	199 (5.6)	119 (7.2)	1,443 (8.1)	265 (7.9)	555 (12.2)	347 (6.7)	155 (5.2)	121 (7.0)
Smoking status, *n* (%)												
Never smoker	3,958 (19.3)	756 (22.9)	718 (15.6)	955 (15.4)	936 (23.2)	593 (25.4)	18,122 (80.0)	2,211 (61.6)	3,418 (69.4)	4,944 (81.8)	4,255 (93.2)	3,294 (96.5)
Past smoker	10,286 (50.0)	1,058 (32.1)	1,966 (42.9)	3,362 (54.1)	2,388 (59.2)	1,512 (64.9)	2,407 (10.6)	711 (19.8)	784 (15.9)	616 (10.2)	208 (4.6)	88 (2.6)
Current smoker	6,226 (30.3)	1,485 (45.0)	1,904 (41.5)	1,898 (30.5)	713 (17.7)	226 (9.7)	2,010 (8.9)	666 (18.6)	724 (14.7)	485 (8.0)	104 (2.3)	31 (0.9)
Alcohol consumption, *n* (%)												
None or rarely	5,274 (25.7)	718 (21.7)	942 (20.5)	1,323 (21.2)	1,166 (28.7)	1,125 (47.7)	14,273 (63.1)	1,511 (42.0)	2,450 (49.7)	3,661 (60.4)	3,548 (77.3)	3,103 (90.1)
1–149 g ethanol/week	5,202 (25.3)	1,084 (32.8)	1,100 (23.9)	1,448 (23.3)	993 (24.5)	577 (24.4)	6,308 (27.9)	1,457 (40.5)	1,794 (36.4)	1,858 (30.7)	890 (19.4)	309 (9.0)
150–299 g ethanol/week	4,068 (19.8)	513 (15.5)	887 (19.3)	1,318 (21.2)	971 (23.9)	379 (16.1)	1,198 (5.3)	326 (9.1)	388 (7.9)	348 (5.7)	111 (2.4)	25 (0.7)
300–449 g ethanol/week	2,958 (14.4)	449 (13.6)	769 (16.7)	1,043 (16.7)	517 (12.7)	180 (7.6)	529 (2.3)	187 (5.2)	184 (3.7)	119 (2.0)	34 (0.7)	5 (0.2)
≥450 g ethanol/week	3,050 (14.8)	539 (16.3)	903 (19.6)	1,097 (17.6)	411 (10.1)	100 (4.2)	315 (1.4)	114 (3.2)	116 (2.4)	74 (1.2)	10 (0.2)	1 (0.03)
History of diabetes, *n* (%)	2,108 (10.3)	107 (3.2)	359 (7.8)	764 (12.3)	618 (15.2)	260 (11.0)	1,503 (6.6)	45 (1.3)	189 (3.8)	460 (7.6)	478 (10.4)	331 (9.6)
History of hypertension, *n* (%)	5,432 (26.4)	267 (8.1)	877 (19.1)	1,865 (29.9)	1,535 (37.8)	888 (37.6)	4,878 (21.5)	134 (3.7)	543 (11.0)	1,329 (21.9)	1,529 (33.2)	1,343 (38.8)

^a^The number of conserved blood samples includes extra plasma or serum sampling.

^b^Body mass index is calculated as self-reported body weight (kg) divided by self-reported height (m)^2^.

The number of missing values are as follows: body mass index, 212 men and 404 women; education level, 1,436 men and 2,230 women; household income, 1,809 men and 4,799 women; smoking status, 89 men and 119 women; alcohol consumption, 7 men and 35 women.

Urine albumin concentration and serum creatinine concentration were measured using the latex agglutination method and enzymatic method, respectively. Albuminuria was evaluated using ACR, and estimated GFR (eGFR) was obtained using the following formula: eGFR (mL/min/1.73 m^2^) = 194 × [serum creatinine (mg/dL)]^−1.094^ × [age (years)^−0.287^ × 0.739 (for women)].^19^ Albuminuria and eGFR were classified as follows^20^: <30, 30–299, and ≥300 mg/g; and <30, 30–59, 60–89, and ≥90 mL/min/1.73 m^2^, respectively.

### Follow-up

Follow-up questionnaire surveys are scheduled every 5 years for 20 years from the baseline survey. Follow-up questionnaire items will be based on those used in the baseline survey.

Information on which patients die or move out of the study area will be obtained from the local government. Annual health checkup data will be obtained from the local government or the designated entities. Cancer incidence is being followed using the cancer registry of Niigata Prefecture. Information on any use of social services is being obtained from the local government. Case confirmation and clinical information are based on information mainly obtained from the relevant hospitals.

### Primary outcome measures

The primary outcome is CKD. CKD will be diagnosed by self-reported questionnaire. After baseline assessments, letters of inquiry are distributed and collected annually concerning participants’ health condition, disease history (including renal disease), and visits to medical institutions in the past year. Self-reported information will be verified with relevant medical institutions by our research team. Furthermore, cases of eGFR-based or urine abnormality-based CKD will be identified during health checks every year after baseline assessment. Other study outcomes include all-cause death, cancer incidence, cardiovascular disease incidence, and use of social services due to disability.

### Ethics

Written informed consent was received from all participants. The study protocol was reviewed and approved by the Research Ethics Committee of Niigata University (approval numbers 2012-1640, 2015-2140, and 2017-0054).

## RESULTS

Baseline characteristics for questionnaire respondents are shown in Table 2. The 43,217 participants (age range, 40–105 years) comprised 20,559 men and 22,658 women, with a mean age of 63.3 (SD, 12.5) years in men and 64.3 (SD, 13.3) years in women. The number of participants by study area was 22,792 in Minamiuonuma, 16,971 in Uonuma, and 3,454 in Yuzawa. In total, self-reported mean BMI was 23.1 (SD, 3.0) kg/m^2^ for men and 22.3 (SD, 3.3) kg/m^2^ for women. Absolute mean difference (measured value minus self-reported value) of BMI was 0.04 for men and 0.05 for women. The numbers of participants with a self-reported history of diabetes and hypertension were 3,611 (8.4%) and 10,310 (23.9%), respectively.

Baseline health checkup data or biochemical samples (blood or urine) were obtained for 3,972 men and 4,080 women, with mean ages of 69.0 (SD, 10.6) years and 68.5 (SD, 10.0) years, respectively. Median ACR was 10.0 (interquartile range [IQR], 5.0–24.0) mg/g for men and 13.0 (IQR, 7.7–27.0) mg/g for women (total *n* = 8,024). Median eGFR was 73.6 (IQR, 63.5–84.5) for men and 73.5 (IQR, 64.4–83.5) for women (total *n* = 8,044). Participants with ACR ≥30 mg/g and eGFR <60 mL/min/1.73 m^2^ were 1,741 (21.7%) and 1,361 (16.9%), respectively. Participants with ACR <30 mg/g and eGFR ≥60 mL/min/1.73 m^2^ included 2,672 men and 2,713 women, with mean ages of 66.9 (SD, 10.8) years and 66.3 (SD, 10.0) years, respectively.

Questionnaire participants included 6,945 with biochemical sampling (baseline health-check examination); of these, 3,384 were men and 3,561 were women, with mean ages of 69.4 (SD, 10.2) years and 68.5 (SD, 9.7) years, respectively. Table 3 shows baseline characteristics for these participants, including the distributions of ACR and eGFR levels. Compared with questionnaire participants, biochemical sampling participants were older and had a lower percentage of current smokers.

**Table 3.** Selected baseline characteristics of participants with both questionnaire and biochemical sampling data, by age and sex

	Men Total	Age at baseline, years					Women Total	Age at baseline, years				
	
		40–49	50–59	60–69	70–79	≥80		40–49	50–59	60–69	70–79	≥80
*N*	3,385	183	305	1,069	1,310	518	3,562	174	349	1,292	1,325	422
Residence area at baseline, *n* (%)												
Minamiuonuma City	1,945 (57.5)	113 (61.8)	187 (61.3)	601 (56.2)	761 (58.1)	283 (54.7)	1,913 (53.7)	107 (61.5)	217 (62.2)	689 (53.3)	693 (52.3)	207 (49.2)
Uonuma City	1,210 (35.8)	63 (34.4)	113 (37.1)	435 (40.7)	462 (35.3)	137 (26.5)	1,366 (38.4)	64 (36.8)	118 (33.8)	553 (42.8)	512 (38.6)	119 (28.3)
Yuzawa Town	229 (6.8)	7 (3.8)	5 (1.6)	33 (3.1)	87 (6.6)	97 (18.8)	282 (7.9)	3 (1.7)	14 (4)	50 (3.9)	120 (9.1)	95 (22.6)
Body mass index^a^, *n* (%)												
<18.5 kg/m^2^	164 (4.8)	3 (1.6)	19 (6.2)	44 (4.1)	60 (4.6)	38 (7.3)	337 (9.5)	18 (10.3)	44 (12.6)	116 (9.0)	115 (8.7)	44 (10.5)
18.5–24.9 kg/m^2^	2,510 (74.2)	127 (69.4)	223 (73.1)	791 (74)	962 (73.4)	407 (78.8)	2,506 (70.4)	125 (71.8)	243 (69.6)	920 (71.2)	922 (69.6)	296 (70.3)
≥25.0 kg/m^2^	710 (21.0)	53 (29.0)	63 (20.7)	234 (21.9)	288 (22.0)	72 (13.9)	718 (20.2)	31 (17.8)	62 (17.8)	256 (19.8)	288 (21.7)	81 (19.2)
eGFR^b^, *n* (%)												
<30, mL/min/1.73 m^2^	13 (0.4)	0 (0)	0 (0)	3 (0.3)	7 (0.5)	3 (0.6)	7 (0.2)	0 (0)	0 (0)	1 (0.1)	3 (0.2)	3 (0.7)
30–59, mL/min/1.73 m^2^	601 (17.8)	3 (1.6)	21 (6.9)	106 (9.9)	274 (20.9)	197 (38.0)	530 (14.9)	2 (1.2)	19 (5.4)	122 (9.5)	258 (19.5)	129 (30.6)
60–89, mL/min/1.73 m^2^	2,271 (67.1)	115 (62.8)	203 (66.6)	761 (71.2)	904 (69.0)	288 (55.8)	2,517 (70.7)	121 (69.5)	262 (75.1)	940 (72.8)	928 (70.0)	266 (63.0)
≥90, mL/min/1.73 m^2^	499 (14.8)	65 (35.5)	81 (26.6)	199 (18.6)	125 (9.5)	29 (5.6)	506 (14.2)	51 (29.3)	68 (19.5)	228 (17.7)	136 (10.3)	23 (5.5)
Urine albumin-creatinine ratio, *n* (%)												
<30, mg/g	2,671 (79.1)	175 (95.6)	273 (89.5)	886 (83.0)	991 (75.8)	346 (67.2)	2,773 (78.0)	164 (94.3)	308 (88.5)	1,089 (84.3)	962 (72.8)	250 (59.8)
30–299, mg/g	636 (18.8)	8 (4.4)	30 (9.8)	169 (15.8)	281 (21.5)	148 (28.7)	721 (20.3)	8 (4.6)	38 (10.9)	192 (14.9)	334 (25.3)	149 (35.7)
≥300, mg/g	71 (2.1)		2 (0.7)	13 (1.2)	35 (2.7)	21 (4.1)	60 (1.7)	2 (1.2)	2 (0.6)	11 (0.9)	26 (2.0)	19 (4.6)
Educational level, *n* (%)												
Junior high school	1,564 (49.7)	26 (14.4)	67 (22.5)	473 (45.1)	774 (60.5)	224 (65.5)	1,861 (55.9)	16 (9.2)	59 (17.4)	636 (50.1)	948 (73.7)	202 (77.7)
High school	1,106 (35.1)	90 (50.0)	142 (47.7)	423 (40.3)	384 (30.0)	67 (19.6)	1,022 (30.7)	89 (51.2)	187 (55.2)	459 (36.1)	254 (19.8)	33 (12.7)
Junior college	255 (8.1)	41 (22.8)	63 (21.1)	76 (7.2)	47 (3.7)	28 (8.2)	393 (11.8)	60 (34.5)	82 (24.2)	154 (12.1)	75 (5.8)	22 (8.5)
University or higher	224 (7.1)	23 (12.8)	26 (8.7)	77 (7.3)	75 (5.9)	23 (6.7)	53 (1.6)	9 (5.2)	11 (3.2)	21 (1.7)	9 (0.7)	3 (1.2)
Household income, *n* (%)												
0–2,990,000 yen	1,496 (48.9)	62 (35.0)	116 (40.6)	509 (50.2)	598 (50.8)	211 (52.1)	1,424 (51.5)	76 (46.3)	118 (38.1)	551 (48.9)	560 (58.3)	119 (58.6)
3,000,000–5,990,000 yen	1,140 (37.3)	82 (46.3)	133 (46.5)	370 (36.5)	417 (35.4)	138 (34.1)	956 (34.6)	60 (36.6)	131 (42.3)	429 (38.1)	286 (29.8)	50 (24.6)
6,000,000–8,990,000 yen	255 (8.3)	17 (9.6)	24 (8.4)	90 (8.9)	92 (7.8)	32 (7.9)	237 (8.6)	19 (11.6)	35 (11.3)	96 (8.5)	67 (7.0)	20 (9.9)
≥9,000,000 yen	169 (5.5)	16 (9.0)	13 (4.6)	45 (4.4)	71 (6.0)	24 (5.9)	147 (5.3)	9 (5.5)	26 (8.4)	51 (4.5)	47 (4.9)	14 (6.9)
Smoking status, *n* (%)												
Never smoker	723 (21.5)	38 (20.8)	55 (18.2)	172 (16.2)	303 (23.3)	155 (30.2)	3,117 (87.9)	99 (57.2)	266 (76.2)	1,108 (85.8)	1,241 (94.3)	403 (96.9)
Past smoker	1,849 (55.0)	69 (37.7)	109 (36.0)	581 (54.6)	781 (60.1)	309 (60.1)	269 (7.6)	44 (25.4)	48 (13.8)	111 (8.6)	55 (4.2)	11 (2.6)
Current smoker	792 (23.5)	76 (41.5)	139 (45.9)	311 (29.2)	216 (16.6)	50 (9.7)	159 (4.5)	30 (17.3)	35 (10.0)	72 (5.6)	20 (1.5)	2 (0.5)
Alcohol consumption, *n* (%)												
None or rarely	822 (24.3)	39 (21.3)	70 (23.0)	207 (19.4)	320 (24.5)	186 (36.0)	2,388 (67.1)	84 (48.3)	185 (53.0)	758 (58.7)	994 (75.1)	367 (87.6)
1–149 g ethanol/week	860 (25.4)	57 (31.2)	65 (21.3)	257 (24.0)	340 (26.0)	141 (27.3)	937 (26.3)	59 (33.9)	123 (35.2)	416 (32.2)	292 (22.1)	47 (11.2)
150–299 g ethanol/week	745 (22.0)	23 (12.6)	56 (18.4)	226 (21.1)	325 (24.8)	115 (22.2)	145 (4.1)	18 (10.3)	24 (6.9)	70 (5.4)	29 (2.2)	4 (1.0)
300–449 g ethanol/week	507 (15.0)	30 (16.4)	58 (19.0)	187 (17.5)	183 (14.0)	49 (9.5)	57 (1.6)	7 (4.0)	12 (3.4)	31 (2.4)	6 (0.5)	1 (0.2)
≥450 g ethanol/week	449 (13.3)	34 (18.6)	56 (18.4)	192 (18.0)	141 (10.8)	26 (5.0)	31 (0.9)	6 (3.5)	5 (1.4)	17 (1.3)	3 (0.2)	0 (0)
History of diabetes, *n* (%)	377 (11.1)	4 (2.2)	16 (5.3)	132 (12.4)	166 (12.7)	59 (11.4)	221 (6.2)	1 (0.6)	8 (2.3)	91 (7.0)	96 (7.3)	25 (5.9)
History of hypertension, *n* (%)	1,070 (31.6)	11 (6.0)	43 (14.1)	331 (31.0)	481 (36.7)	204 (39.5)	883 (24.8)	5 (2.9)	26 (7.5)	285 (22.1)	421 (31.8)	146 (34.7)

eGFR, estimated glomerular filtration rate.

^a^Body mass index is calculated as measured body weight (kg) divided by measured height (m)^2^.

^b^eGFR (mL/min/1.73 m^2^) = 194 × [serum creatinine (mg/dL)] − 1.094 × [age (years) − 0.287 × 0.739 (for women)].

The numbers of missing values are as follows: eGFR, one woman; urine albumin-to-creatinine ratio, 6 men and 7 women; education level, 235 men and 232 women; household income, 324 men and 797 women; smoking status, 20 men and 16 women; alcohol consumption, 1 man and 3 women.

## DISCUSSION

Here, we report baseline profiles and the prevalence of albuminuria and low eGFR among participants in the Uonuma CKD cohort study, for which registration is now complete.

Three other large cohort studies of CKD are being conducted in Europe, North America, and Asia.^8^^,^^9^^,^^21^ For example, the Prevention of Renal and Vascular End-Stage Disease (PREVEND) study^21^ in Europe is a population-based prospective cohort study designed to examine the natural course of microalbuminuria and its relationship with renal and cardiovascular disease in the general population. The United States CRIC study^8^ was designed to examine risk factors for the progression of chronic renal insufficiency and its relationship to cardiovascular disease, but it enrolled only subjects with eGFR 20–70 mL/min/1.73 m^2^. The KNOW-CKD study,^9^ recently initiated in Korea, targets patients with various stages of CKD. The PREVEND study is a large-scale but not primarily Asian study, and the CRIC and KNOW-CKD studies are hospital-based, focusing mainly on patients as a target population and end-stage renal disease or cardiovascular disease as an outcome. Conversely, the Uonuma CKD cohort study targets community-based residents and assesses outcomes including CKD and other lifestyle-related diseases. Recently, a population-based prospective cohort study, Healthy Life in an Urban Setting (HELIUS), was started in the Netherlands.^22^ While CKD is assessed in HELIUS, it is not a primary outcome.

The Uonuma CKD cohort study is also unique in terms of exposure measures. We have collected data on wide-ranging lifestyle and environmental factors in order to provide useful information for primary prevention of CKD. In summary, our study has the following strengths: first, this is the first population-based cohort study of CKD in Asia and is being conducted on a large scale. Second, this study is designed to comprehensively clarify CKD risk factors and assess their interactions, using both lifestyle and environment information and biochemical specimens. We particularly focus on early-stage CKD as an outcome, which has not been done previously. Finally, the study is planned to last 20 years, which will enable us to clarify the natural history of CKD.

This study also has some limitations. First, because the study is being conducted in the Uonuma area of Japan, the results may not be generalizable to other Japanese populations or other ethnicities worldwide. Also, baseline analysis of urine and blood was done only once, likely causing misclassification of albuminuria and uncertainty regarding chronicity. Though this study obtained a reasonably high response rate, the self-reported questionnaire participants account for the majority of the study population. Thus, it will be important to validate the accuracy of the self-reported information in the future.

### Conclusions

The Uonuma CKD cohort study examines the impact of lifestyle on CKD development and progression. The goal is to provide evidence for preventing the onset and progression of CKD in order to decrease the individual and social burden of this condition.
